# Amplifying Flutamide Sensing through the Synergetic Combination of *Actinidia*-Derived Carbon Particles and WS_2_ Platelets

**DOI:** 10.1021/acsomega.4c02795

**Published:** 2024-06-26

**Authors:** Yiran Luo, P. Rupa Kasturi, Tara N. Barwa, Eithne Dempsey, Carmel B. Breslin

**Affiliations:** † Department of Chemistry, Maynooth University, Maynooth, Co. Kildare W23 F2H6, Ireland; ‡ Kathleen Lonsdale Institute, Maynooth University, Maynooth, Co. Kildare W23 F2H6, Ireland

## Abstract

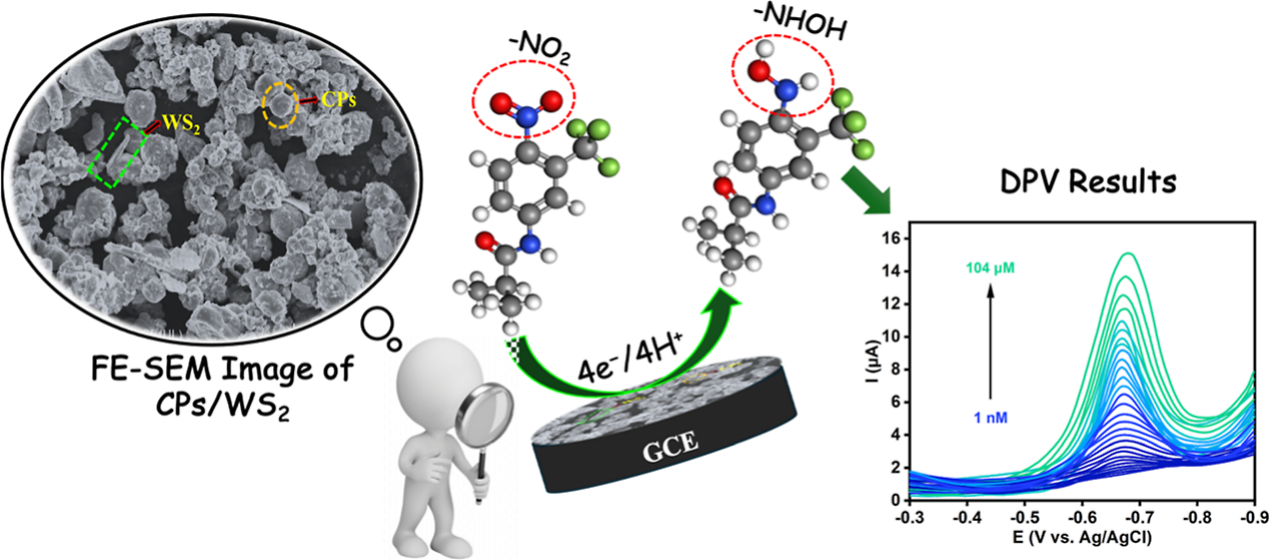

The development of electrochemical sensors for flutamide detection is a crucial step in biomedical research and environmental monitoring. In this study, a composite of *Actinidia*-derived carbon particles (CPs) and tungsten disulfide (WS_2_) was formed and used as an electrocatalyst for the electrochemical detection of flutamide. The CPs had an average diameter of 500 nm and contained surface hydroxyl and carbonyl groups. These groups may help anchor the CPs onto the WS_2_ platelets, resulting in the formation of a CPs-WS_2_ nanocomposite with a high surface area and a conducting network, enabling electron transfer. Using the CPs-WS_2_ composite supported at a glassy carbon electrode, a linear concentration range extending from 1 nM to 104 μM, a limit of detection of 0.74 nM, and a sensitivity of 26.9 ± 0.7 μA μM^–1^ cm^–2^ were obtained in the detection of flutamide in a phosphate buffer. The sensor showed good recovery, ranging from 88.47 to 95.02%, in river water samples, and exhibited very good selectivity in the presence of inorganic ions, including Al^3+^, Co^2+^, Cu^2+^, Fe^3+^, Zn^2+^, NO_3_^–^, SO_4_^2–^, CO_3_^2–^, and Cl^–^.

## 1 Introduction

In recent years, various carbon-based structures and nanostructures, such as carbon dots,^1^ carbon nanoparticles,^2^ graphene,^3^ carbon fibers and carbon black,^4^ and carbon nanotubes,^5^ have attracted considerable attention. This is not surprising due to their interesting electronic and physiochemical properties, combined with a high surface area and impressive electrical conductivity. Consequently, they are finding increasing applications in electrochemical-based systems, including sensors^6,7^ and energy storage devices.^8,9^ They are readily functionalized with groups, such as −OH and −COOH, which in turn can provide active or coordination sites that can be exploited in the development of sensing materials.^10,11^ Another attractive property is their ability to form composites or hybrids with other materials giving new hybrids that exhibit synergetic effects.^12^

In terms of carbon-based composites, the transition metal dichalcogenides (TMDs) are interesting companion materials. TMDs are a family of layered materials and are represented as MX_2_, where M represents a transition metal and X is a chalcogen atom, normally S or Se.^13^ The M atoms are sandwiched between two chalcogen atoms, with covalent bonding between the M and X atoms, to give stacked layers that are held by weak van der Waals forces. MoS_2_ is a well-known member of this family, finding a wide range of applications.^14,15^ Indeed, carbon-based materials have been combined with MoS_2_.^16^ Tungsten disulfide, WS_2_, although less well-known, has been employed in the development of electrochemical-based sensors,^17−19^ indicating that it is suitably conducting for sensing applications. In terms of its combination with carbon-based materials, it has been combined with diamond nanoparticles and used in the sensing of food additives^12^ and with carbon nanotubes for the detection of perfluorooctanoic acid.^18^ Therefore, in this paper, carbon particles (CPs) were combined with WS_2_ platelets to form a conducting composite material for the electroanalysis and determination of flutamide (FLD).

Flutamide, 4-nitro-3-trifluoromethyl-isobutylaniline, was selected as it has been widely used to effectively control the growth and spread of prostate cancer,^20^ and it is also employed in the treatment of polycystic ovary syndrome.^21^ However, with its significant use in the healthcare sector, and its ineffective removal from wastewater treatment plants, it has been detected in the aquatic ecosystem.^22^ Anticancer drugs, such as FLD, can be extremely toxic, and this is alarming in terms of the health of aquatic species, impacting on their reproductive endocrine systems.^23^ FLD sensors have previously been reported, including sensors fabricated from different oxide phases, such as nickel oxides,^24^ molybdenum oxides,^25^ and cobalt oxides,^26−29^ and various sensors that employ graphene or reduced graphene oxide.^30−37^ However, the sensing of FLD with WS_2_ or any WS_2_-based composite material has not been reported.

In this study, we show that CPs synthesized from kiwi juice, with an average diameter of 500 nm, form a stable composite with WS_2_ platelets, when the size of the particles and platelets are matched. The resulting composite is very effective in the electrochemical detection of FLD. This new composite, CPs-WS_2_, has a high surface area and good electrical conductivity, and it enables the electrochemical detection of nM concentrations of FLD.

## 2 Experimental Section

### 2.1 Reagents and Chemicals

All the chemicals used in this study were of analytical grade and were sourced from Sigma-Aldrich/Merck. Bulk WS_2_ powders, tannic acid, flutamide (FLD), [Fe(CN)_6_]^3–/4–^, HCl, NH_4_NO_3_, NaCl, KCl, CO(NH_2_)_2_, CaCl_2_, MgNO_3_, Na_2_SO_4_, K_2_HPO_4_, and KH_2_PO_4_ were among the chemicals used. To synthesize CPs, *Actinidia deliciosa*, commonly known as golden kiwi fruit, was purchased from Dunnes, a local Irish supermarket. The fruit was carefully selected based on its freshness and quality and then thoroughly washed with deionized water (DI) and dried.

### 2.2 Instruments and Measurements

Various analytical techniques were conducted in this study to investigate the properties of the composites formed. Fourier transform infrared spectroscopy (FTIR) measurements were carried out using a Nicolet iS50 FTIR spectrometer, while a Cary 50 spectrometer was used to record UV–visible data. Surface morphology was monitored using high-resolution scanning electron microscopy (HR-SEM) with a Regulus 8100 field emission Hitachi microscope. Raman spectroscopy [inVia Reflex Raman Microscope (532 and 632.8 nm)] and powder X-ray diffraction (XRD) (Shimadzu XRD-7000) were also employed. These techniques were used to provide insights into the chemistry of the composites, thereby contributing to an understanding of their electrochemical potential as sensing materials.

Electrochemical measurements included cyclic voltammetry (CV), differential pulse voltammetry (DPV), and electrochemical impedance spectroscopy (EIS). The CV measurements were conducted using a Solartron 1287 potentiostat, and EIS studies were performed with a Solartron 1287 potentiostat coupled with a 1255 FRA (Solartron). DPV measurements were carried out by recording the current response using a CHI440 CH Instruments, Inc. potentiostat. All potentials are reported relative to the Ag/AgCl scale.

### 2.3 Synthesis of CPs

**Scheme 1:**
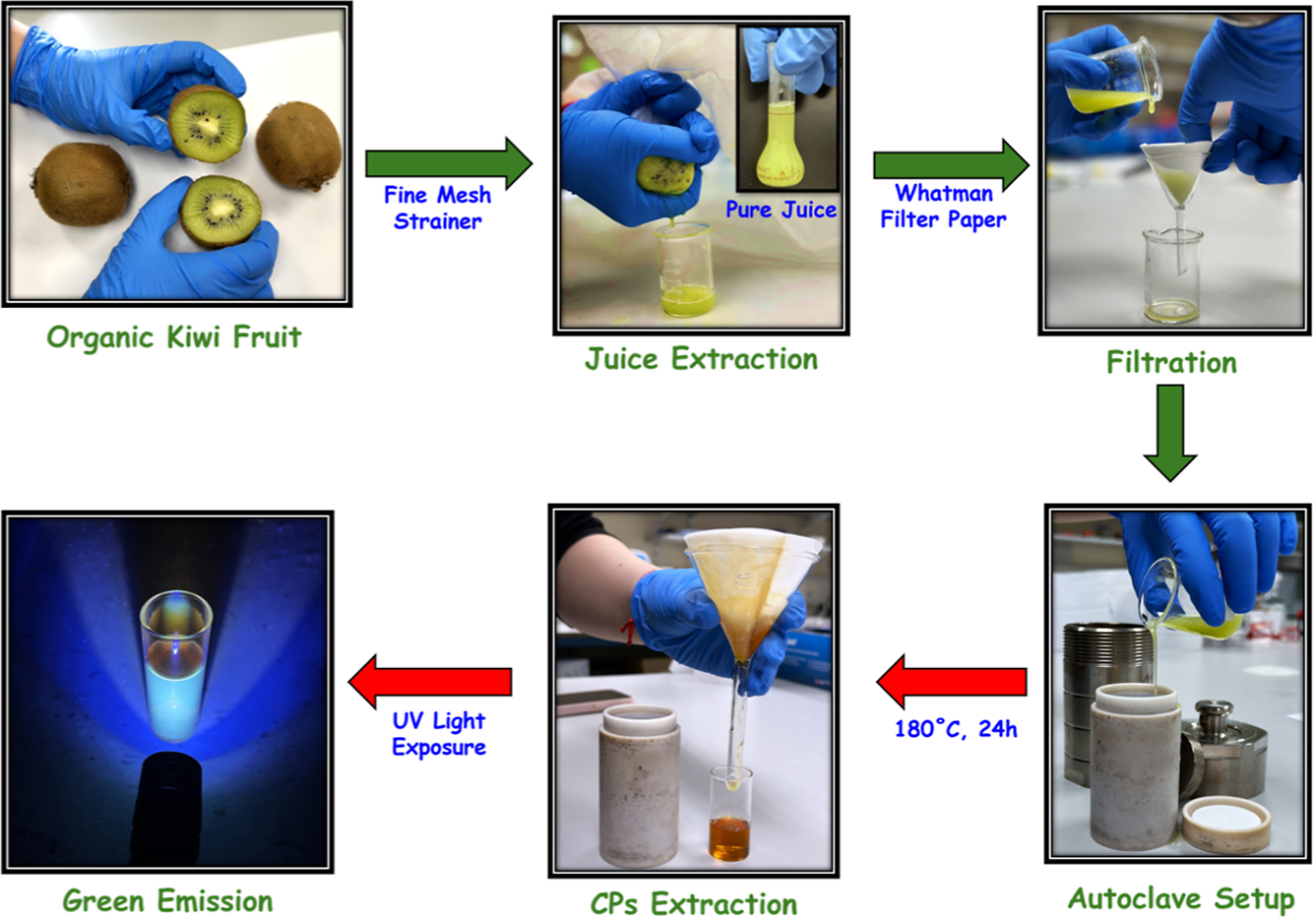
Synthetic Method Used to Prepare the CPs

To prepare CPs from *A. deliciosa*, the methodology developed for the preparation of carbon nanostructures using sustainable approaches was adapted.^38^ The fruit (fresh fruit, used on the day it was purchased from the supermarket) was first washed with DI, dried, and peeled. The peeled fruit was cut into small pieces and blended using a laboratory blender. The resulting puree was then squeezed through cheesecloth to obtain the concentrated juice. The juice, 40 mL, was transferred to a hydrothermal autoclave (100 mL) and heated to 180 °C for 24 h, as shown in Scheme 1. The hydrothermal product was collected, filtered at least three times to remove the larger carbon structures, and finally centrifuged to obtain a clear solution of the CPs. The collected solution containing the CPs was refrigerated at 4 °C for future use. Two additional samples were prepared by combining the blended puree with either water or ethanol in a 3:1 ratio prior to the hydrothermal reaction. The resulting CPs were labeled as CPs (concentrated juice), CPs-DI (juice diluted with DI), and CPs-EtOH (juice diluted with ethanol).

### 2.4 Synthesis of CPs-WS_2_

To prepare the composites comprising the CPs and WS_2_, 1 g of WS_2_ powder was mixed with 10 mL of each of the CPs synthesized in Section 2.3 and obtained from the *A. deliciosa* juice, CPs, CPs-DI, and CPs-EtOH. The mixture was thoroughly homogenized and centrifuged for 20 min at 2000 rpm. This was repeated 5 times, with each round of centrifugation being followed by washing of the composite with DI. The composite was then dried overnight at 60 °C to remove any residual moisture. By combining WS_2_ with the different types of CPs, three distinct composite materials were obtained, namely, the CPs combined with the concentrated juice, CPs-WS_2_, CPs-DI-WS_2_, and CPs-EtOH-WS_2_, with all the materials adopting silver/black metallic-like colors.

### 2.5 Preparation and Use of the Glassy Carbon Electrode (GCE)/CPs-WS_2_ Sensor

The FLD sensor was fabricated using the prepared CPs-WS_2_ composites. First, a GCE (3 mm) was polished on a microcloth (Aka-Napel) with a diamond suspension (Akasol, 1 μm sized particles). The GCE was then rinsed with DI and dried in an air stream. The CPs-WS_2_ composites were sonicated in DI (1 mg/1 mL) for 30 min. Then, 5 μL of this dispersion was drop-cast onto the GCE followed by its drying at room temperature, resulting in the formation of a modified GCE with the CPs-WS_2_ composite material evenly spread over the electrode surface. A standard three-electrode cell was used for the electrochemical measurements, comprising the GCE/CPs-WS_2_, Ag/AgCl reference, and a high surface area Pt wire, which served as the counter electrode. All solutions were deoxygenated with nitrogen for at least 30 min before the electrochemical tests.

The optimized sensor was applied in the sensing of FLD in both real river water samples and artificial human urine. The water samples were collected from the local canal in Maynooth. The samples were filtered immediately to remove debris and then stored at 4 °C until tested (with all tests completed within 4 h). For these real water measurements, both the GCE and carbon screen-printed electrodes (SPE), obtained from Metrohm, were used to support the CPs-WS_2_ composite. The artificial urine media with a pH of 6.0 was prepared by combining HCl (0.02 M), NH_4_NO_3_ (0.85 g), NaCl (14.1 g), KCl (2.8 g), CaCl_2_ (0.6 g), Mg(NO_3_)_2_·*x*(H_2_O) (0.43 g), Na_2_SO_4_ (0.42 g), and CO(NH_2_)_2_ (17.3 g) in DI to give a final volume of 80 mL.

## 3 Results and Discussion

### 3.1 Characterization of CPs and the CPs-WS_2_ Composite

**Figure 1:**
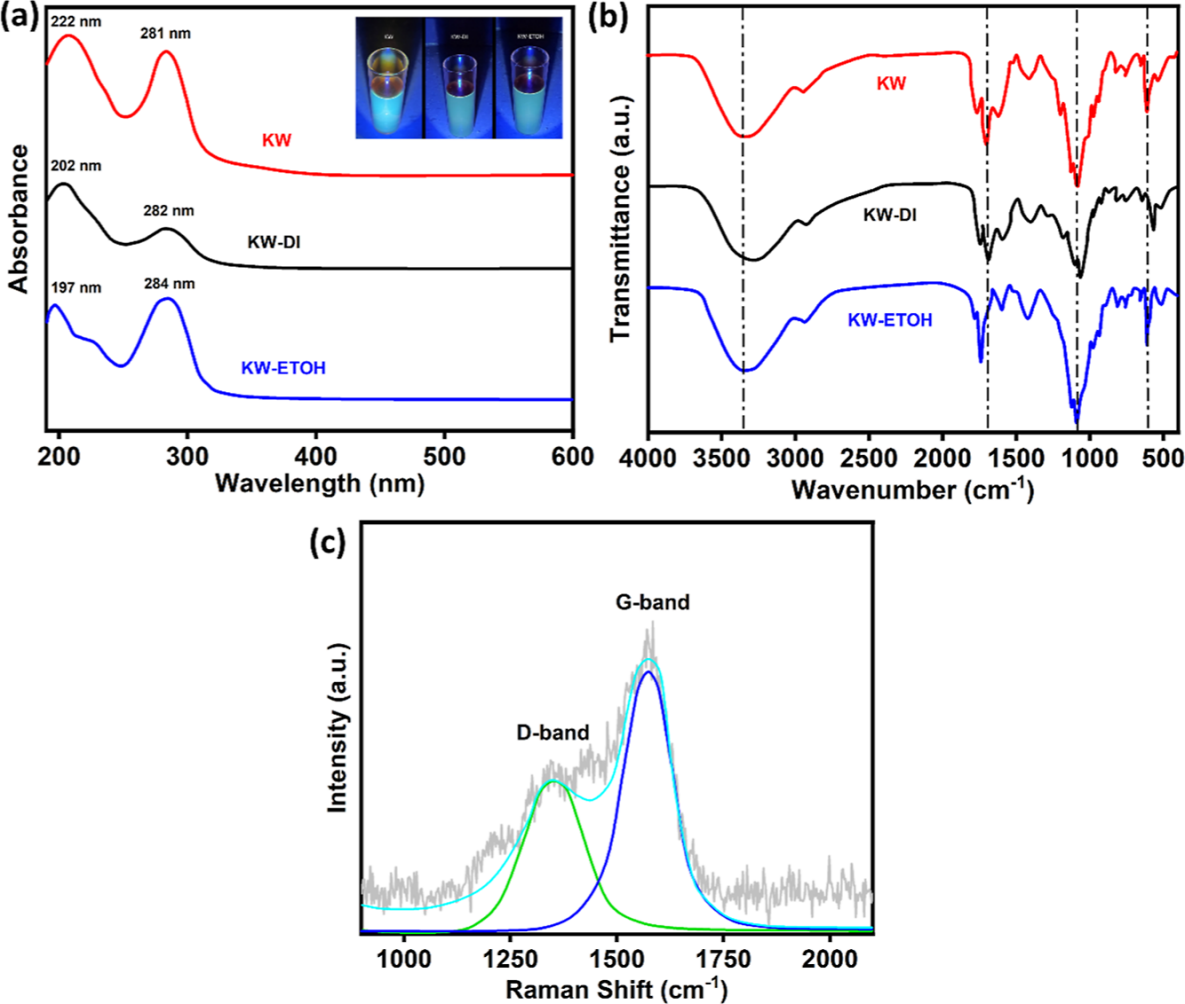
(a) UV–vis absorption spectra recorded for CPs, CPs-DI, and CPs-EtOH; (b) FTIR spectra recorded for CPs, CPs-DI, and CPs-EtOH; (c) Raman spectrum recorded for CPs.

**Figure 2:**
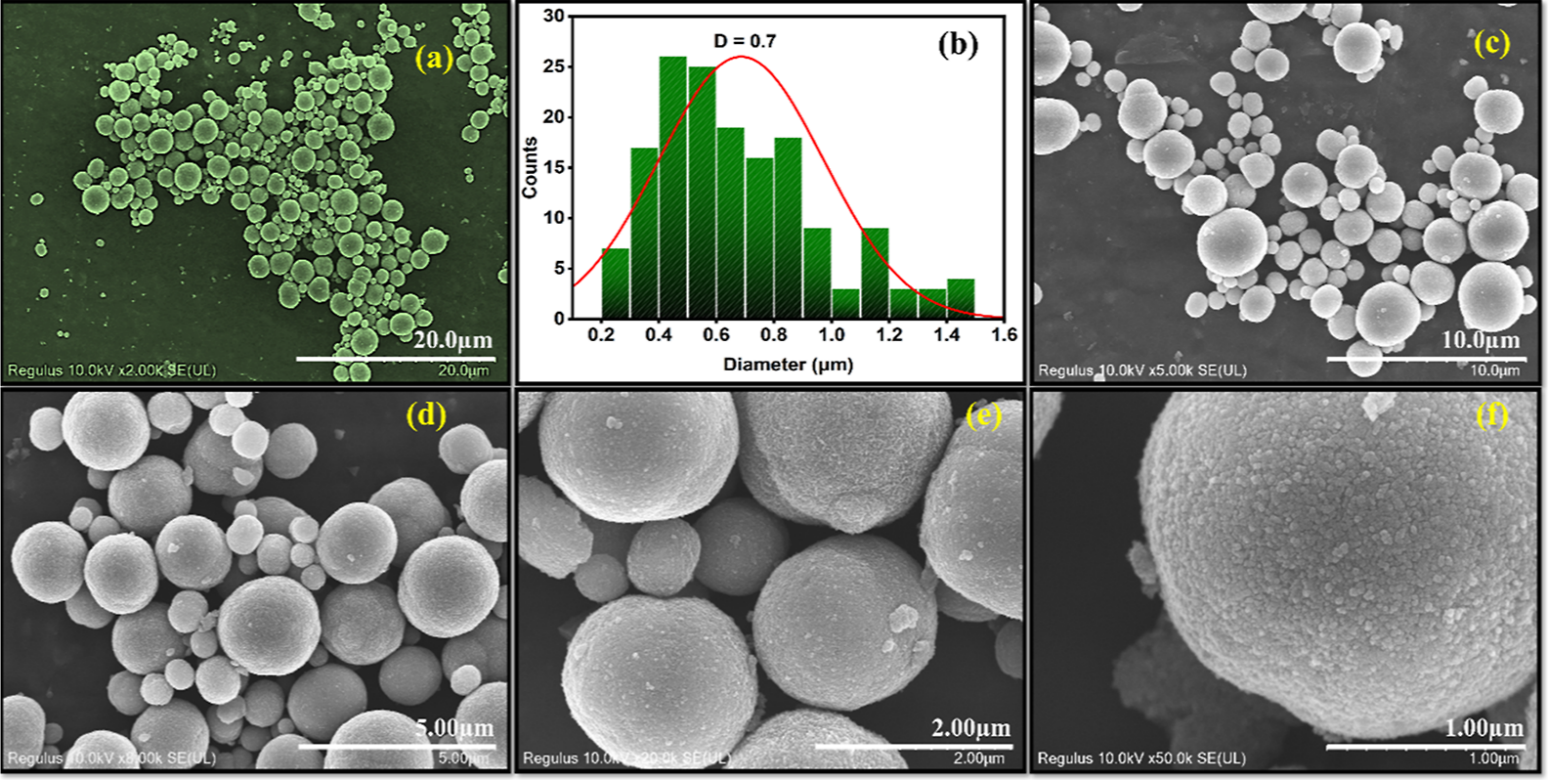
(a) SEM micrographs recorded for CPs at low resolution; (b) size summary of CPs from (a); SEM micrographs recorded for the CPs at different magnifications with scales at (c) 10; (d) 5; (e) 2; (f) 1 μm.

Once the CPs were synthesized, they were characterized using a combination of UV–visible, FTIR, and HR-SEM, and the relevant data are presented in Figures 1 and 2. In Figure 1a,b, the UV–visible and FTIR spectra are shown for the three CPs, and the nature of the extracted juice clearly influences the spectra. The CPs show strong absorption in the ultraviolet region with two prominent peaks for all three CPs in the vicinity of 200 and 280 nm. These can be attributed to the higher energy π–π* transition of the C=C bonds and the lower energy *n*–π* transition of the C=O bonds.^39^ There are no absorbance bands from about 350–600 nm and no evidence of polymer chains that typically absorb at these longer wavelengths. For the higher energy absorption peak, the λ_max_ values vary from 222 to 197 nm, indicating variations in the nature or concentration of the surface groups. The FTIR spectra, depicted in Figure 1b, show four main peaks. The broad peak at about 3350 cm^–1^ indicates the presence of O–H. Strong peaks are evident at 1640 and 1023 cm^–1^, and these can be attributed to the presence of C=O and C–O groups, while the peak at about 600 cm^–1^ may indicate the presence of C–H groups. This is consistent with the presence of O–H, C=O, and C–H moieties at the surface of the CPs. There is also a peak at 1405 cm^–1^, which is indicative of C=C, and suggests that the CPs possess a graphitic structure, which is very relevant in terms of electrochemical-based sensing.

The Raman spectrum of the CPs is presented in Figure 1c and exhibits the D and G bands, characteristic of carbon-based materials. The D band at about 1350 cm^–1^ is linked to carbon defects, while the G-band is related to the sp^2^-hybridized carbon network. The *I*_D_/*I*_G_ ratio was computed as 0.75, indicating some defects in the CPs.

The size and morphology of the CPs were studied using HR-SEM, and representative micrographs are presented in Figure 2. The size distribution histogram indicates that the CPs vary from approximately 0.2–1.5 μm in diameter, with the highest population ranging in size from about 0.4–0.6 μm, Figure 2b. The surface morphology of the CPs is more evident in Figure 2e,f. The larger CPs appear to be composed of much smaller CPs, or carbon quantum dots, assembled and connected. The variation in size of the CPs, Figure 2b, may indicate a progressive-like nucleation and growth of the particles. Alternatively, the growth may proceed through an Ostwald ripening mechanism whereby the smaller CPs, or carbon dots, dissolve and redeposit onto the larger forming CPs. Indeed, Ostwald ripening has previously been observed during the vapor phase growth of carbon spheres.^40^

**Figure 3:**
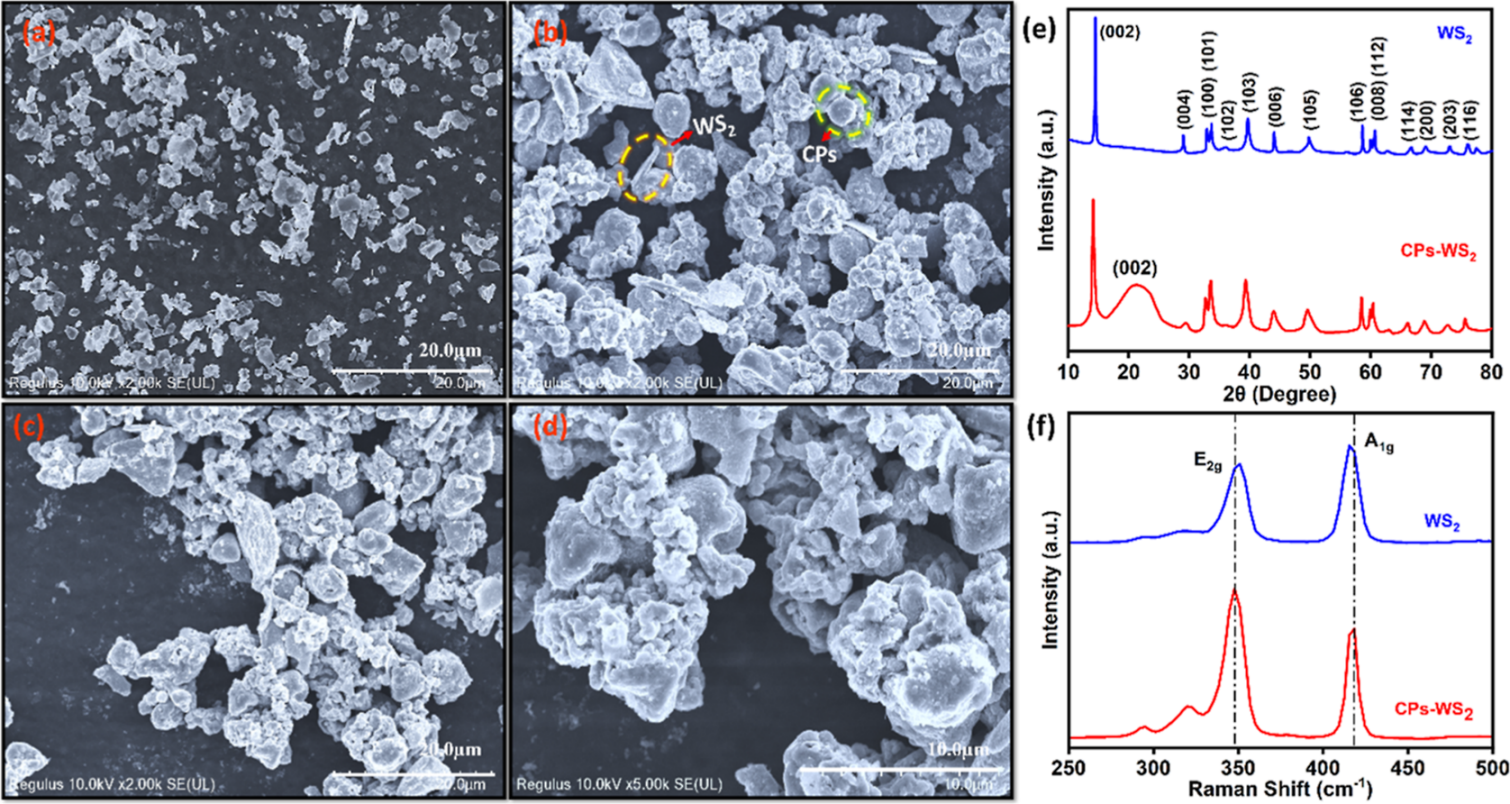
SEM images for (a) bulk WS_2_; (b) CPs-WS_2_; (c) CPs-WS_2_; (d) CPs-WS_2_; (e) XRD patterns for WS_2_ and CPs-WS_2_; (f) Raman spectra for WS_2_ and CPs-WS_2_.

In Figure 3, SEM, XRD, and Raman data are shown for the CPs-WS_2_ composite. The WS_2_ appears as platelets of different sizes, ranging from 0.2 to 1.5 μm, Figure 3a, with some as large as 2 μm, making them ideal supports for the CPs, with diameters between 0.4 and 0.8 μm. Indeed, this can be clearly seen in Figure 3b–d, where the CPs are anchored to the WS_2_ platelets. This composite has a very different morphology compared to either the CPs, Figure 2, or the WS_2_, Figure 3a, providing a high surface area that is appealing in the development of electrochemical-based sensors. Clear evidence for the formation of this CPs-WS_2_ composite is seen on comparing the XRD and Raman spectra recorded for WS_2_ and CPs-WS_2_. The XRD pattern of WS_2_, with diffraction angles at 14.3, 28.9, 32.8, 33.5, 35.8, 39.5, 43.9, 49.7, 58.5, 59.8, 60.4, 62.6, 66.5, 69.1, 72.8, 75.8, and 77.2° corresponding to the (002), (004), (100), (101), (102), (103), (006), (105), (106), (008), (102), (122), (114), (102), (200), (203), and (116) planes, are evident. These are in good agreement with previous publications.^41^ When the CPs-WS_2_ composite is formed, the diffraction pattern changes, with the diffraction peaks associated with the WS_2_ becoming somewhat broader. In addition, a broad peak corresponding to the (002) plane of the CPs is evident, indicating that the CPs are amorphous in nature due to the presence of the functional groups.^39^ Similarly, the Raman data show changes in the WS_2_ spectrum on adding the CPs. The plot recorded for the WS_2_ is in good agreement with previous reports.^42^ However, on forming the composite, a different spectrum was observed, as shown in Figure 3f. The ratio of the E_2g_/A_1g_ peak heights varies from 0.83 for pure WS_2_ to 1.83 for the CPs-WS_2_ composite. Furthermore, the position of the E_2g_ peak shifts from 350.9 to 347.5 cm^–1^, while A_1g_ changes from 415.5 to 418.5 cm^–1^ when combined with the CPs. These data clearly show the formation of a new composite that is distinct from the individual components, when the CPs and WS_2_ are combined.

### 3.2 Electrochemical Properties and Detection of FLD at GCE/CPs-WS_2_

The CPs, CPs-DI, and CPs-EtOH were combined with the WS_2_ to give the composites, CPs-WS_2_, CPs-DI-WS_2_, and CPs-EtOH-WS_2_, and were then tested in the electrochemical reduction of 100 μM FLD. Peak currents of 8.242, 6.864, and 7.520 μA with peak potentials of −680, −702, and −762 mV were obtained for CPs-WS_2_, CPs-DI-WS_2_, and CPs-EtOH-WS_2_, respectively. This shows that the CPs-WS_2_ composite provides not only the highest peak current but also the more thermodynamically favorable reduction potential. Therefore, CPs-WS_2_ was selected for all further studies and named as GCE/CPs-WS_2_ when drop-cast onto the GCE. The drop-casting method was further studied to determine the optimum drop-casting volume and layers of drop-casting solution. The drop-casting volume was varied from 1 to 10 μL, and the corresponding sensor was evaluated in the electrochemical reduction of 100 μM FLD using CV. The influence of volume is seen in Figure S1a (Supporting Information), and clearly 5 μL gives the optimum coverage of the GCE. This 5 μL volume was then repeatedly applied to the GCE followed by room temperature drying between the applications, and the data are summarized in Figure S1b, where a single layer provides the best detection of FLD. Accordingly, a single 5 μL volume of the CPs-WS_2_ dispersion was applied in all studies.

#### 3.2.1 Electrochemical Characteristics

**Figure 4:**
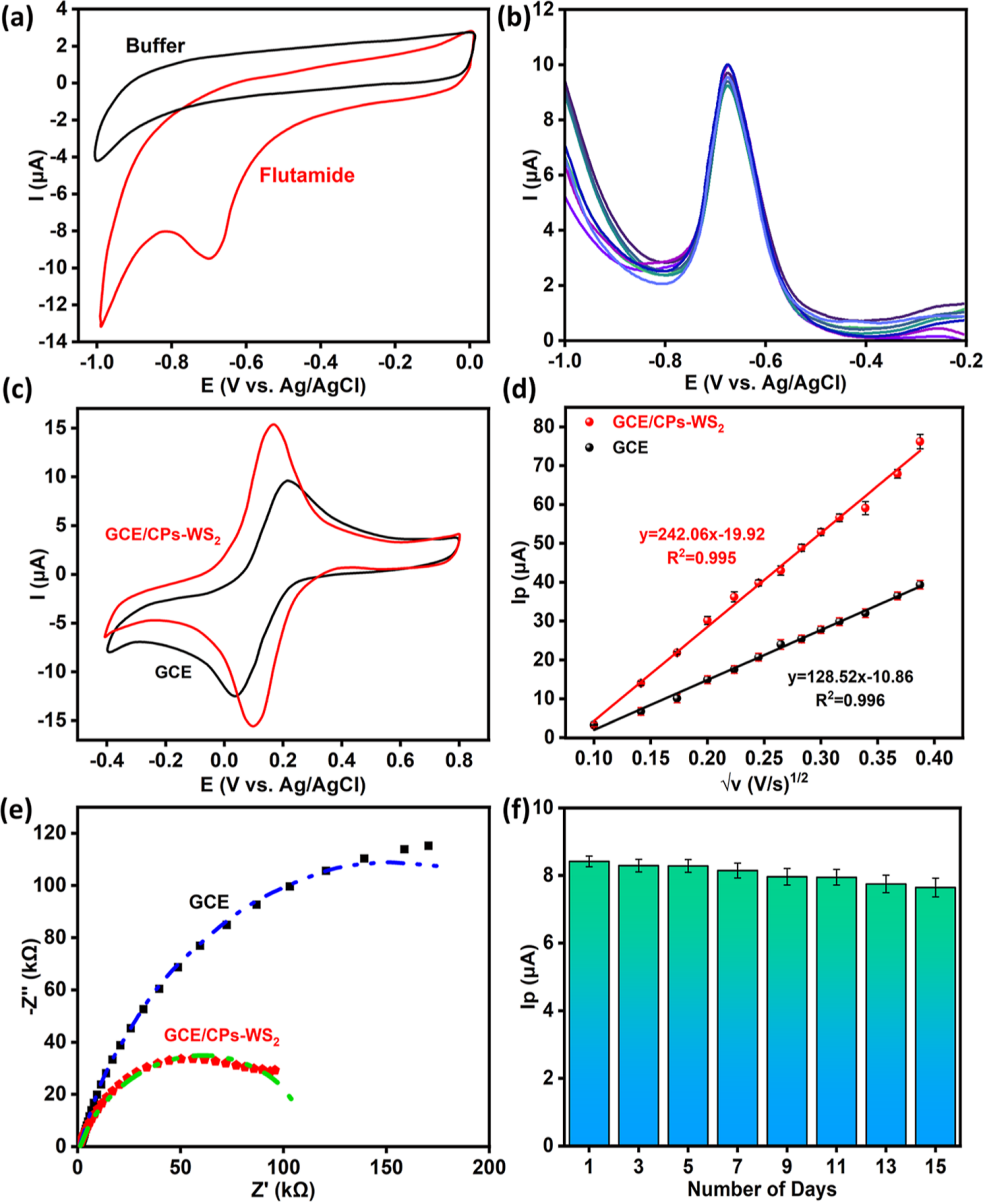
(a) CVs for GCE/CPs-WS_2_ at pH 7.0 in phosphate buffer in the presence and absence of 100 μM FLD; (b) DPVs recorded for 9 repeated experiments for GCE/CPs-WS_2_ in 100 μM FLD in phosphate buffer; (c) CVs recorded and (d) peak current plotted vs square root of scan rate for GCE/CPs-WS_2_ and GCE in 2.5 mM [Fe(CN)_6_]^3–/4–^ dissolved in 0.1 M NaCl; (e) EIS spectra for GCE and GCE/CPs-WS_2_ at −0.68 V in buffered 100 μM FLD; (f) stability of GCE/CPs-WS_2_ over 15 days with peak current in 100 μM FLD in phosphate buffer plotted as a function of immersion period in phosphate buffer.

**Scheme 2:**
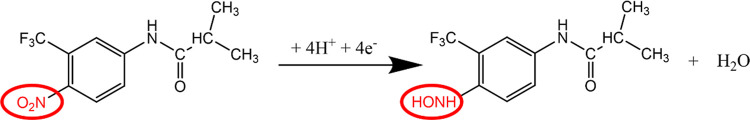
Irreversible Reduction of FLD, with the Reduction of the −NO_2_ to the −NHOH Group with the Transfer of 4e^–^/4H^+^

The electrochemical reduction of FLD at the GCE/CPs-WS_2_-modified sensor was initially studied using cyclic voltammetry (CV), and a representative CV recorded at 50 mV s^–1^ is illustrated in Figure 4a. In the absence of the FLD, a low background current is observed with no evidence of any redox reactions. On adding 100 μM FLD, a clear reduction wave at −0.68 V, corresponding to the reduction reaction highlighted in Scheme 2, occurs, indicating that GCE/CPs-WS_2_ facilitates the irreversible electrochemical reduction of FLD. The capacitive characteristics of GCE/CPs-WS_2_ are evident in Figure 4a, with the relatively high background currents. This is consistent with the high surface area morphology of CPs-WS_2_, Figure 3. The performance of the GCE/CPs-WS_2_-modified sensor is compared with that of GCE/CPs and GCE/WS_2_ in Table 1, where the peak current recorded in 100 μM FLD and the reproducibility are summarized. While the GCE gives relatively good reproducibility, the peak current is low at 4.1 μA. When the GCE was modified with exfoliated WS_2_ sheets, where the exfoliation was performed with sonication for 30 min in 0.5 mM tannic acid, a higher peak current was achieved, but the reproducibility was low. Likewise, poor reproducibility with an RSD of 22.48% was achieved with the CPs, possibly due to their agglomeration when exposed to the aqueous solution. However, when the CPs-WS_2_ composites were employed, improved reproducibility was seen, and higher peak currents were achieved. It is also evident that the ratio of WS_2_ to CPs impacts the detection of FLD, with the best detection, in terms of enhanced interelectrode reproducibility (RSD of 0.90%) and peak current (8.24 μA for 100 μM FLD), obtained when higher amounts of WS_2_ were used. The data in Table 1 also show that very good precision is achieved for GCE/CPs-WS_2_, with the RSD at 0.90% for three sensors, fabricated on different days.

**Table 1:** Peak Current Recorded at 50 mV s^–1^ for 100 μM FLD in 0.1 M Phosphate Buffer with a pH of 7.0 for the Various Modified Electrodes

modified electrode	experiment I current (μA)	experiment II current (μA)	experiment III current (μA)	average current (μA)	RSD (%)
GCE	4.109	4.096	4.116	4.107	0.25
GCE/ta-WS_2_	6.162	5.711	5.487	5.786	5.94
GCE/CPs	4.755	4.304	3.012	4.023	22.48
GCE/CPs-WS_2_ (0.5 g WS_2_/10 mL CPs)	7.714	7.204	6.941	7.286	5.39
GCE/CPs-WS_2_ (1.0 g WS_2_/10 mL CPs)	8.286	8.154	8.279	8.240	0.90

The repeatability of the GCE/CPs-WS_2_ sensor in the detection of 100 μM FLD is shown in Figure 4b, where the DPV experiments were repeated nine times. An average peak current of 8.13 μA, with a maximum current of 8.27 μA, was obtained and the RSD was computed as 0.90%, indicating very good repeatability. This indicates very good intraelectrode repeatability, which is important in terms of the sensor stability and suggests it could be employed for repeated use.

As shown in Figure 3, CPs-WS_2_ provides a high surface area morphology, and accordingly, the surface area of the GCE/CPs-WS_2_ was estimated using the well-known [Fe(CN)_6_]^3–/4–^ probe. Cyclic voltammograms were recorded at different scan rates in 2.5 mM [Fe(CN)_6_]^3–/4–^ dissolved in 0.1 M NaCl. Typical cyclic voltammograms recorded for GCE and GCE/CPs-WS_2_ are shown in Figure 4c, where more reversible-like behavior and higher peak currents are seen with GCE/CPs-WS_2_. The peak-to-peak separation was computed as 71 mV for GCE/CPs-WS_2_ compared to a much higher value of 184 mV for the GCE. This indicates a more efficient electron transfer step in the electrochemical conversion between the [Fe(CN)_6_]^3–^ and [Fe(CN)_6_]^4–^ ions at GCE/CPs-WS_2_. The peak currents for the oxidation waves are plotted in Figure 4d as a function of the square root of the scan rate. Good linearity is achieved, with the linear regression equations deduced as *I*_p_ (μA) = 242.06 ± 5.10 *v*^1/2^ (V^1/2^ s^–1/2^) – 19.92 ± 1.38, (*R*^2^ = 0.9951, adjusted (adj.) *R*^2^ = 0.9946) for the GCE/CPs-WS_2_ and *I*_p_ (μA) = 128.52 ± 2.30 *v*^1/2^ (V^1/2^ s^–1/2^) – 10.86 ± 0.63 (*R*^2^ = 0.9964, adj. *R*^2^ = 0.9961) for the GCE. The Randles–Sevick equation, eq 1, was employed to estimate the *A* values. Here, *I*_p_ is the peak current, *C* represents the concentration of the [Fe(CN)_6_]^3–/4–^ probe, *D* is the diffusion coefficient of the probe, *n* corresponds to the number of electrons transferred, *v* gives the scan rate, and *A* is the electroactive surface area. Using this approach, the *A* values were estimated as 0.069 ± 0.002 cm^2^ and 0.130 ± 0.003 cm^2^ for the GCE and GCE/CPs-WS_2_, respectively, corresponding to a 1.6-fold increase in the electroactive area on forming the modified GCE/CPs-WS_2_ electrode.
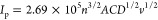
1

To gain information on the conducting properties of GCE/CPs-WS_2_ in the presence of FLD, EIS was employed and compared with the GCE. These data were recorded between 100 kHz and 7 mHz using a perturbation potential of 10 mV and are displayed in Figure 4e. The plots are characterized by a semicircle at the higher frequencies with evidence of a diffusional process emerging at 7 mHz. Clearly, GCE/CPs-WS_2_ has a lower impedance with a smaller semicircle and presents the more conducting interface. In these plots, the symbols indicate the experimental data, while continuous traces represent the simulated profiles. For GCE/CPs-WS_2_, a simple Randles cell proved suitable in the fitting of the experimental data. In this case, the circuit consists of a solution resistance term, *R*_s_, in series with a constant phase element, (*Q*_dl_), and charge transfer resistance (*R*_CT_), in parallel, eq 2.
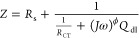
2

The *R*_CT_ values were computed as 293 ± 3.0 and 120 ± 6.0 kΩ for the GCE and GCE/CP-WS_2_, respectively, indicating a more conducting GCE/CPs-WS_2_. The impedance data recorded for GCE/CPs and GCE/WS_2_ are shown in the Supporting Information, Figure S2, where the individual components of the composite have a higher *R*_CT_, with larger diameters for the depressed semicircles. Indeed, the *R*_CT_ was determined as 313 ± 10.0 kΩ for GC/CPs, while GC/WS_2_ exhibited a clear diffusional term with a Warburg resistance of 505 ± 6.0 kΩ. The enhanced conducting properties of the CPs-WS_2_ composite are clear from this analysis.

In Figure 4e, the overall stability of GCE/CPs-WS_2_ is summarized. These data were recorded by storing the GCE/CPs-WS_2_ electrodes in the phosphate buffer at room temperature over a 15 day storage period. The electrodes were removed at different intervals, and the DPVs were recorded in the presence of 100 μM FLD. As shown in the figure, good long-term stability is achieved. The peak current decreased only slightly from 8.24 to 8.02 μA over the 15 day period, indicating a loss of only 0.95% in the peak current over the 15 days. A similar analysis was performed with 10, 30, and 50 μM FLD, and these data are summarized in Table S1. Again, good stability is seen with the RSD over the 15 days computed as 1.38, 1.87, and 5.64% for the 50, 30, and 10 μM FLD, respectively.

#### 3.2.2 Influence of the pH and Surface and Diffusional-Controlled Processes

**Figure 5:**
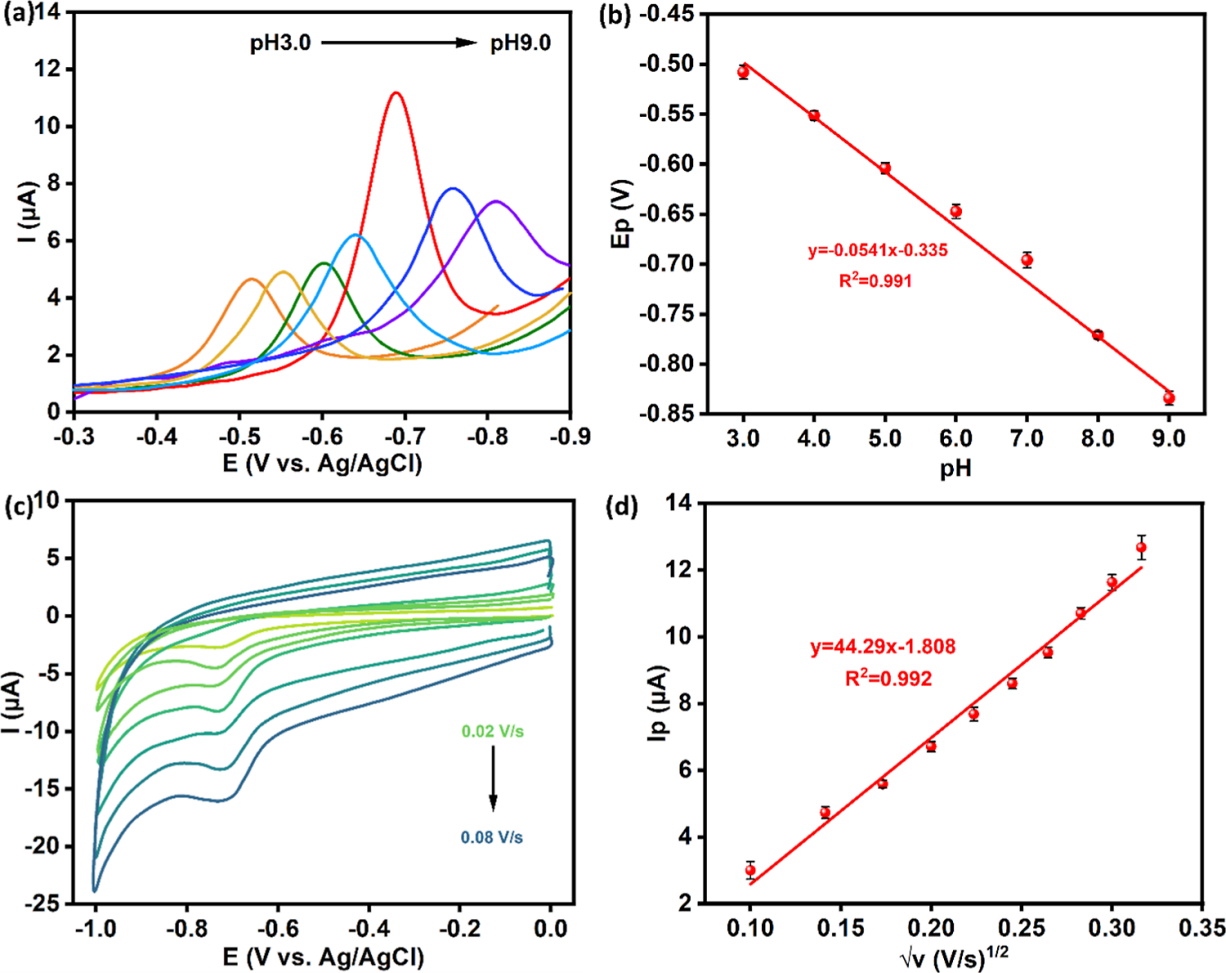
(a) DPVs for 100 μM FLD at pH values of 3.0, 4.0, 5.0, 6.0, 7.0, 8.0, and 9.0; (b) plot of peak potential, *E*_p_, as a function of pH; (c) CV curves at different scan rates in a 100 μM FLD solution, pH of 7.0; (d) peak current, *I*_p_, plotted as a function of the square root of the scan rate (*v*).

The influence of pH on the electrochemical reduction of FLD is summarized in Figure 5a,b. The DPV voltammograms show that both the peak current and peak potential are dependent on the pH of the solution. The peak potentials shift from −0.50 to −0.84 V as the pH of the solution is increased from 3.0 to 9.0. It is also evident from Figure 5a that the maximum peak current is seen at a pH of 7.0. The peak current decreases as the pH of the solutions is increased from 7.0 to 9.0 and lowered from 7.0 to 3.0. On plotting the peak potential as a function of pH, a linear plot was achieved with a slope of 0.0541 ± 0.0147 V pH^–1^, which is close to the value predicted from the Nernst equation at 298 K of 0.0591 V pH^–1^. This is consistent with the reduction mechanism detailed in Scheme 2, indicating the transfer of equal numbers of electrons and hydrogen ions.

Another important parameter in the reduction of FLD at the GCE/CPs-WS_2_ is the way the FLD reaches and interacts with the electrode surface. Therefore, to determine if the reduction of FLD was under diffusion or adsorption control, scan rate studies were performed, and the resulting CV curves are shown in Figure 5c. The capacitive behavior of the GCE/CPs-WS_2_ is evident in these CVs. As the scan rate is increased, the peak current increases. There is no indication that the peak potential shifts to lower potentials with increasing scan rates, which is indicative of sluggish electron transfer reactions. Instead, the peak potentials remain relatively constant. In Figure 5d, the peak current is shown plotted against the square root of the scan rate, consistent with eq 3, corresponding to a diffusion-controlled process for an irreversible process. Here *n*_α_ is the number of electrons transferred prior to and including the rate-determining step. A linear plot was obtained with a regression equation, *I*_p_ (μA) = 44.29 ± 1.95 *v*^1/2^ (V^1/2^ s^–1/2^) – 1.808 ± 0.45 (*R*^2^ = 0.9922, adj. *R*^2^ = 0.9825), which is consistent with a diffusion-controlled reduction of FLD. However, a linear plot [*I*_p_ (μA) = 100.3 ± 4.5 *v* (V s^-1^) + 2.61 ± 0.64, (*R*^2^ = 0.9894, adj. *R*^2^ = 0.9785)] was also obtained when the peak current was plotted as a function of scan rate, consistent with adsorption control, eq 4. In this adsorption relationship, *F*, *R,* and *T* have their usual meanings, α is the charge transfer coefficient, *A* represents the surface area, and τ* corresponds to the surface-bound redox couple.
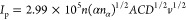
3
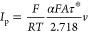
4

To clearly distinguish between these two processes, the logarithm of the peak current was plotted against the logarithm of the scan rate. The corresponding plot is depicted in Figure S3, with a linear equation of log *I*_p_ (*I*_p_/μA) = 0.552 ± 0.04 log *v* (V s^–1^) + 1.624 ± 0.4, (*R*^2^ = 0.9894, adj. *R*^2^ = 0.9815). The slope of this plot at approximately 0.5 clearly indicates a diffusion-controlled reduction reaction.

### 3.3 Sensing Performance

**Figure 6:**
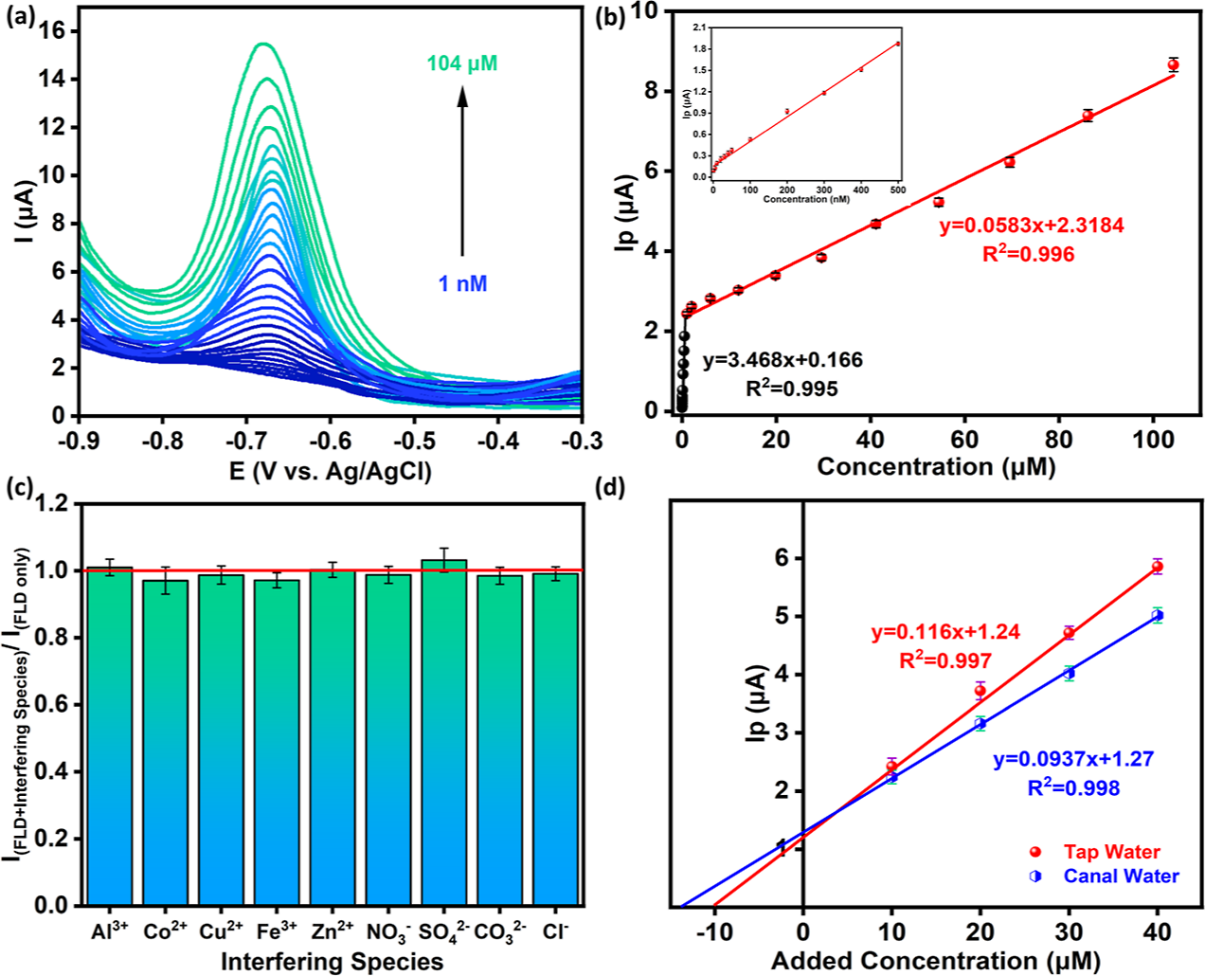
(a) DPVs for concentrations from 1 nM to 104 μM, (b) calibration curve with the DPV peak current shown against the FLD concentration, (c) ratio of peak current with and without the interferent for 100 μM FLD and 1 mM interferent, and (d) standard addition method for the analysis of tap and river water samples spiked with FLD using screen-printed electrodes (SPE/CPs-WS_2_).

The performance of GCE/CPs-WS_2_ in the sensing of FLD was evaluated using a combination of sensitivity, selectivity, and real sample analyses. In Figure 6a, the DPVs recorded as a function of the concentration of FLD in phosphate buffer are shown, while the DPVs obtained in 1, 5, 10, 20, and 30 nM FLD are provided in Figure S4. As the FLD has a limited solubility in water, the highest concentration employed in this analysis was 104 μA. As shown in Figure 6a, the peak current increases with increasing FLD concentrations. The peak potential shifts slightly to more negative values from −674 mV at 20 nM FLD to −688 mV for 104 μA. However, the most significant aspect of this composite is the well-defined reduction wave obtained at a concentration of 1 nM, Figure S4, making this sensor suitable for the sensing of nM concentrations of FLD.

The peak currents from the DPVs were plotted as a function of the concentration of the FLD to give the calibration curve, as depicted in Figure 6b. Two linear regions are seen. The linear calibration curve equation, *I*_p_ (μA) = 0.0583 ± 0.001 *C* (μM) + 2.3184 ± 0.08 (*R*^2^ = 0.9960, adj. *R*^2^ = 0.9945), was obtained for concentrations ranging from 1 to 104 μM. For lower concentrations extending from 1 to 500 nM, shown in the inset and in Figure S5, the linear regression equation, *I*_p_ (μA) = 3.468 ± 0.076 *C* (μM) + 0.166 ± 0.016 (*R*^2^ = 0.9950, adj. *R*^2^ = 0.9945), was achieved. Using the well-known expression for the limit of detection (LOD), where the LOD = 3σ/sensitivity, the LOD was calculated as 0.74 nM, using the lower concentration linear range. This compares well with the fully defined peak obtained for 1 nM FLD, Figure S4. The sensitivity was computed as 26.7 μA μM^–1^ cm^–2^ using the estimated electroactive surface area of 0.130 cm^2^ for the GCE/CPs-WS_2_ and the lower concentration range from 1 to 500 nM. These analytical characteristics are compared with some of the recently reported sensors for FLD in Table 2. The linear range is similar to many of these previously reported sensors. However, the LOD is considerably lower than many of these reports, while the high sensitivity of 26.7 ± 0.7 μA μM^–1^ cm^–2^ is a clear benefit in the detection of the lower nM concentrations. Furthermore, the well-defined reduction wave observed for a 1 nM concentration, Figure S4, is an obvious advantage of this GCE/CPs-WS_2_ material in the detection of low concentrations of FLD (see ).

**Table 2:** Comparison of GCE/CPs-WS_2_ with Recently Reported Sensors for FLD

sensor	method	linear range (μA)	LOD (nM)	sensitivity (μA μM^–1^ cm^–2^)	refs
GCE/CPs-WS_2_	DPV	0.001–104	0.74	26.7	this work
GCE/CaMoO_4_/MoO_3_	*I*–*t*	0.0449–1184	1.6	53.34	(25)
MoW–P/RGO	DPV	0.3–1152	9	0.50	(35)
BiVO_4_rGO/CE-BN/GCE	DPV	0.04–102	11	3.80	(43)
Sr@FeNi–S/GnRbs		0.020–0.450	20		(44)
ZnO–Co_3_O_4_@C_3_N_4_	DPV	0.01–98.6	3.7	5.33	(27)
Sn–ZnO/rGO	DPV	0.01–170	7.3	14.10	(34)
AuNP@rGO/PPyNT	DPV	0.01–1214	2.5	50.06	(31)
GOS/CaTiO_3_	*I*–*t*	0.015–1184	5.7	1.073	(30)
β-Cu_2_V_2_O_7_/PC	*I*–*t*	0.01–2.11	0.62	24.33	(45)
MoS_2_–CeZrO	DPV	0.019–668	5	0.353	(46)

**Table 3:** Comparison Table of Different Analysis Samples for Flutamide

samples		added drug FLD (μM)	recovery (%)	RSD (%)
water analysis	tap water	10	90.2	3.70
		30	94.6	2.23
		50	97.3	1.07
		100	98.8	0.75
	canal/river water	10	88.5	5.27
		30	91.1	3.43
		50	94.5	2.35
		100	95.0	1.14
biological analysis	artificial urine	10	89.5	5.70
		30	92.7	4.09
		50	95.4	2.28
		100	96.8	1.04

The selectivity of GCE/CPs-WS_2_ was studied using ions that are commonly found in real water samples. For these studies, 1 mM of the interferent was added to 100 μM FLD to give a 10-fold interferent concentration. The ratio of the peak current obtained for the pure 100 μM FLD to the 100 μM FLD with 1 mM interferent was calculated, and these data are summarized in Figure 6c. The addition of these ions has little effect on the detection of FLD, with the ratio of the peak currents, remaining very close to unity. The selectivity of the GCE/CPs-WS_2_ sensor was probed further using real water samples, collected from a local canal, and artificial urine samples. The results from these studies are summarized in Table 3. The recovery varies from 90.2 to 98.8% for tap water, 88.5 to 95.0% for canal water, and 89.5 to 96.8% for the artificial urine samples. The highest RSD values were 3.70% for tap water, 5.27% for canal water, and 5.70% for the artificial urine at the lower concentrations of 10 μM FLD. These data show acceptable recovery in all the real samples. To explore further the versatility of the CPs-WS_2_ dispersions, SPE were modified with these dispersions, and a standard addition method was used with both tap water and the canal/river water samples. The CPs-WS_2_ dispersions were applied to the SPE and processed using the optimized method developed for the GCE. The water sample was initially spiked with 10 μM FLD to form the FLD sample. Further additions of FLD were then added, and the DPVs were recorded to determine the peak currents. These data are shown in Figure 6d. The linear regression equations were determined as *I*_p_ (μA) = 0.116 ± 0.003 *C* (μM) + 1.24 ± 0.09 (*R*^2^ = 0.9978, adj. *R*^2^ = 0.9968) for the tap water and *I*_p_ (μA) = 0.094 ± 0.002 *C* (μM) + 1.27 ± 0.04 (*R*^2^ = 0.9984, adj. *R*^2^ = 0.9978) for the canal water samples. The concentration of the initial FLD sample was determined as 10.6 μM, giving an error of 6%, for the tap water sample, indicating acceptable detection in this aquatic system. However, the FLD was computed at a concentration of 13.5 μM for the canal/river water sample, indicating that the presence of contaminants in the real water sample make the detection of FLD more complex. This can also be seen in the linear plots, with the gradients varying from 0.116 μA μM^–1^ for the tap water to 0.094 μA μM^–1^ for the canal water samples.

The accuracy of the GCE/CPs-WS_2_ sensor was evaluated using the standard addition method with DI as the aqueous component. A known concentration of FLD was added to the sample, standard additions were made, and the computed concentration from the linear plot was compared with the known concentration added. A typical plot is shown in Figure S6, where the known concentration of FLD was 20 μM. Using the linear plot, the concentration was computed as 20.2 μM, corresponding to a 1% error, indicating very good accuracy.

## 4 Summary and Conclusions

In summary, GCE/CPs-WS_2_ can serve as a highly sensitive and selective sensor for the electrochemical analysis of FLD, giving a well-defined redox wave in the presence of 1 nM FLD. A clear synergetic effect is seen between the WS_2_ and CPs as their sizes and dimensions facilitate these interactions, and this results in a high surface area and conducting material, suitable for electrochemical-based applications. GCE/CPs-WS_2_ shows impressive stability, very good selectivity, sensitivity, and acceptable recovery in complex canal/river water and artificial urine samples. Therefore, the GCE/CPs-WS_2_ sensor has real potential in the determination of FLD in both environmental and biomedical applications.

## Supporting Information Available

Volume of the CPs-WS_2_ dispersion and layers of the dispersion; impedance data for GCE, GCE/CPs, GCE/WS_2_, and GCE/CPs-WS_2_; scan rates for GCE/CPs-WS_2_; DPVs at nM concentrations; calibration curve from 1 to 500 nM; accuracy measurement; and long-term stability (PDF)

## References

[ref1] Molla A.; Youk J. H. Recent progress on electroanalytical sensing of small molecules and biomolecules using carbon dots: A review. J. Ind. Eng. Chem. 2023, 127, 62–81. 10.1016/j.jiec.2023.07.037.

[ref2] Asadian E.; Ghalkhani M.; Shahrokhian S. Electrochemical sensing based on carbon nanoparticles: A review. Sens. Actuators, B 2019, 293, 183–209. 10.1016/j.snb.2019.04.075.

[ref3] Yan Y.; Gong J.; Chen J.; Zeng Z.; Huang W.; Pu K.; Liu J.; Chen P. Recent advances on graphene quantum dots: From chemistry and physics to applications. Adv. Mater. 2019, 31, 1808283 10.1002/adma.201808283. 30828898

[ref4] Krishnan S. K.; Singh E.; Singh P.; Meyyappan M.; Nalwa H. S. A review on graphene-based nanocomposites for electrochemical and fluorescent biosensors. RSC Adv. 2019, 9, 8778–8881. 10.1039/C8RA09577A. 35517682 PMC9062009

[ref5] Schroeder V.; Savagatrup S.; He M.; Lin S.; Swager T. M. Carbon nanotube chemical sensors. Chem. Rev. 2019, 119, 599–663. 10.1021/acs.chemrev.8b00340. 30226055 PMC6399066

[ref6] Breslin C. B.; Branagan D.; Garry L. M. Electrochemical detection of Cr(VI) with carbon nanotubes decorated with gold nanoparticles. J. Appl. Electrochem. 2019, 49, 195–205. 10.1007/s10800-018-1259-2.

[ref7] Branagan D.; Breslin C. B. Electrochemical detection of glucose at physiological pH using gold nanoparticles deposited on carbon nanotubes. Sens. Actuators, B 2019, 282, 490–499. 10.1016/j.snb.2018.11.089.

[ref8] Collins G.; Kasturi P. R.; Karthik R.; Shim J.-J.; Sukanya R.; Breslin C. B. Mesoporous carbon-based materials and their applications as non-precious metal electrocatalysts in the oxygen reduction reaction. Electrochim. Acta 2023, 439, 141678 10.1016/j.electacta.2022.141678.

[ref9] Gao T.; Luo W.; Yang Y.; Zhou Y.; Xu J.; Li N.; Li J.; Liu Z. Engineering hierarchically porous carbon nanorods electrode materials for high performance zinc ion hybrid supercapacitors. Colloids Surf., A 2024, 684, 133057 10.1016/j.colsurfa.2023.133057.

[ref10] Qi H.; Teng M.; Liu M.; Liu S.; Li J.; Yu H.; Teng C.; Huang Z.; Liu H.; Shao Q.; Umar A.; Ding T.; et al. Biomass-derived nitrogen-doped carbon quantum dots: highly selective fluorescent probe for detecting Fe^3+^ ions and tetracyclines. J. Colloid Interface Sci. 2019, 539, 332–341. 10.1016/j.jcis.2018.12.047. 30594008

[ref11] Padmapriya A.; Thiyagarajan P.; Devendiran M.; Kalaivani R. A.; Shanmugharaj A. M. Electrochemical sensor based on N,P–doped carbon quantum dots derived from the banana flower bract (Musa acuminata) biomass extract for selective and picomolar detection of dopamine. J. Electroanal. Chem. 2023, 943, 117609 10.1016/j.jelechem.2023.117609.

[ref12] Blanco E.; Hristova L.; Martínez-Moro R.; Vázquez L.; Ellis G. J.; Sánchez L.; del Pozo M.; Petit-Domínguez M. D.; Casero E.; Quintana C. A 2D tungsten disulphide/diamond nanoparticles hybrid for an electrochemical sensor development towards the simultaneous determination of sunset yellow and quinoline yellow. Sens. Actuators, B 2020, 324, 128731 10.1016/j.snb.2020.128731.

[ref13] Sukanya R.; da Silva Alves D. C.; Breslin C. B. Review—Recent developments in the applications of 2D transition metal dichalcogenides as electrocatalysts in the generation of hydrogen for renewable energy conversion. J. Electrochem. Soc. 2022, 169, 064504 10.1149/1945-7111/ac7172.

[ref14] Alves D.; Kasturi P. R.; Collins G.; Barwa T. N.; Ramaraj S.; Karthik R.; Breslin C. B. 2D layered double hydroxides and transition metal dichalcogenides for applications in the electrochemical production of renewable hydrogen. Mater. Adv. 2023, 4, 6478–6497. 10.1039/D3MA00685A.

[ref15] Luo Y.; Barwa T. N.; Herdman K.; Dempsey E.; Breslin C. B. Electroanalysis of metronidazole using exfoliated MoS_2_ sheets and electrodeposited amorphous MoS_x_. Electrochim. Acta 2023, 462, 142778 10.1016/j.electacta.2023.142778.

[ref16] Luo Y.; Barwa T. N.; Dempsey E.; Karthik R.; Shim J. J.; Sukanya R.; Breslin C. B. Electrochemical detection of sulfanilamide using tannic acid exfoliated MoS_2_ nanosheets combined with reduced graphene oxide/graphite. Environ. Res. 2024, 248, 118391 10.1016/j.envres.2024.118391. 38309562

[ref17] Abid K.; Iannazzo D.; Celesti C.; Khaskhoussi A.; Foti A.; Maalej R.; Gucciardi P. G.; Neri G. A novel 2D-GO@WS_2_ electrochemical platform for the determination of thiram fungicide. J. Environ. Sci. 2024, 136, 226–236. 10.1016/j.jes.2022.11.018. 37923433

[ref18] Mashkoor F.; Mashkoor R.; Shoeb M.; Anwer A. H.; Jeong H.; Jeong C. Freestanding WS_2_-MWCNT nanocomposite for electrochemical detection of contaminants of emerging concern–perfluorooctanoic acid “A forever chemical” and supercapacitor applications. ACS Sustain. Chem. Eng. 2023, 11, 13306–13319. 10.1021/acssuschemeng.3c02376.

[ref19] Jeevanandham G.; Kuppuswamy G. P.; Sesu D. C.; Vediappan K. Screen printed carbon electrode modified with WS_2_ nanosheet incorporated with cobalt oxide for non-enzymatic detection of lactic acid. Surface. Interfac. 2023, 40, 103097 10.1016/j.surfin.2023.103097.

[ref20] Crawford E. D.; Schellhammer P. F.; McLeod D. G.; Moul J. W.; Higano C. S.; Shore N.; Denis L.; Iversen P.; Eisenberger M. A.; Labrie F. Androgen receptor targeted treatments of prostate cancer: 35 years of progress with antiandrogens. J. Urol. 2018, 200, 956–966. 10.1016/j.juro.2018.04.083. 29730201

[ref21] Goodman N. F.; Cobin R. H.; Futterweit W.; Glueck J. S.; Legro R. S.; Carmina E. American association of clinical endocrinologists, American college of endocrinology, and androgen excess and pcos society disease state clinical review: Guide to the best practices in the evaluation and treatment of polycystic ovary syndrome - Part 1. Endocr. Pract. 2015, 21, 1291–1300. 10.4158/EP15748.DSC. 26509855

[ref22] Gouveia T. I. A.; Mota I. H.; Silva A. M. T.; Alves A.; Santos M. S. F. Are cytostatic drugs in surface waters a potential threat?. Sci. Total Environ. 2022, 853, 158559 10.1016/j.scitotenv.2022.158559. 36087660

[ref23] Bhatia H.; Kumar A.; Ogino Y.; Du J.; Gregg A.; Chapman J.; Mclaughlin M. J.; Iguchi T. Effects of the commercial antiandrogen flutamide on the biomarkers of reproduction in male Murray rainbowfish (Melanotaenia fluviatilis). Environ. Toxicol. Chem. 2014, 33, 1098–1107. 10.1002/etc.2524. 24453069

[ref24] Fathi Z.; Jahani S.; Foroughi M. M. Electrode material fabricated by doping holmium in nickel oxide and its application in electrochemical sensor for flutamide determination as a prostate cancer drug. Monatsh. Chem. 2021, 152, 757–766. 10.1007/s00706-021-02794-8.

[ref25] Nataraj N.; Chen T.-W.; Chen S.-M.; Kokulnathan T.; Ahmed F.; Alshahrani T.; Arshi N. Electrochemical detection of anti-cancer drug flutamide in biological fluids with calcium molybdate/molybdenum oxide as an effective electrocatalyst. J. Taiwan Inst. Chem. Eng. 2024, 156, 105348 10.1016/j.jtice.2024.105348.

[ref26] Devi R. K.; Muthusankar G.; Chen S.-M.; Gopalakrishnan G. In situ formation of Co_3_O_4_ nanoparticles embedded N-doped porous carbon nanocomposite: a robust material for electrocatalytic detection of anticancer drug flutamide and supercapacitor application. Microchim. Acta 2021, 188, 196 10.1007/s00604-021-04860-8. 34036435

[ref27] Umesh N. M.; Antolin Jesila J.; Wang S.-F.; Govindasamy M.; Alshgari R. A.; Ouladsmane M.; Asharani I. V. Fabrication of highly sensitive anticancer drug sensor based on heterostructured ZnO-Co_3_O_4_ capped on carbon nitride nanomaterials. Microchem. J. 2021, 167, 106244 10.1016/j.microc.2021.106244.

[ref28] Durai L.; Gopalakrishnan A.; Badhulika S. A low-cost and facile electrochemical sensor for the trace-level recognition of flutamide in biofluids using large-area bimetallic NiCo_2_O_4_ micro flowers. New J. Chem. 2022, 46, 3383–3391. 10.1039/D1NJ05246B.

[ref29] Ravele T.; Hlongwa N. W.; Nkambule T. T. I.; Gumbi N. N.; Sekhosana K. E. Electrochemical sensors based on manganese and cobalt oxide nanostructures for the detection of flutamide and its derivatives in real water samples. J. Cluster Sci. 2024, 35, 285–297. 10.1007/s10876-023-02474-z.

[ref30] Tseng T.-W.; Rajaji U.; Chen T.-W.; Chen S.-M.; Huang Y.-C.; Mani V.; Irudaya Jothi A. Sonochemical synthesis and fabrication of perovskite type calcium titanate interfacial nanostructure supported on graphene oxide sheets as a highly efficient electrocatalyst for electrochemical detection of chemotherapeutic drug. Ultrason. Sonochem. 2020, 69, 105242 10.1016/j.ultsonch.2020.105242. 32673961

[ref31] Sangili A.; Vinothkumar V.; Chen S.-M.; Veerakumar P.; Lin K.-C. Gold nanoparticle embedded on a reduced graphene oxide/ polypyrrole nanocomposite: Voltammetric sensing of furazolidone and flutamide. Langmuir 2020, 36, 13949–13962. 10.1021/acs.langmuir.0c02448. 33174747

[ref32] Mondal S.; Sharma P. K. Voltammetric determination of anti-cancer drug flutamide using Ag/N-doped reduced graphene oxide and its analytical application in pharmaceutical formulations. Appl. Phys. A: Mater. Sci. Process. 2024, 130, 41 10.1007/s00339-023-07188-7.

[ref33] Sharma T. S. K.; Hwa K.-Y. Rational design and preparation of copper vanadate anchored on sulfur doped reduced graphene oxide nanocomposite for electrochemical sensing of antiandrogen drug nilutamide using flexible electrodes. J. Hazard. Mater. 2021, 410, 124659 10.1016/j.jhazmat.2020.124659. 33279323

[ref34] Hwa K.-Y.; Santhan A.; Tata S. K. S. Fabrication of Sn-doped ZnO hexagonal micro discs anchored on rGO for electrochemical detection of anti-androgen drug flutamide in water and biological samples. Microchem. J. 2021, 160, 105689 10.1016/j.microc.2020.105689.

[ref35] Karuppusamy N.; Subburaj S.; Chen S. M.; Veerakumar P.; Lin K.-Y.; Meenakshi S. Determination of flutamide toward a real-time electrochemical sensor based on ultrathin reduced graphene oxide-covered MoW-P. New J. Chem. 2023, 47, 18671–18681. 10.1039/D3NJ02800C.

[ref36] Jiang L.-L.; Niu X.; Pei W.-Y.; Ma J.-F. Electrochemical detection of flutamide by the composite of complex based on thiacalix[4]arene derivatives and reduced graphene oxide. Inorg. Chem. 2023, 62, 12803–12813. 10.1021/acs.inorgchem.3c01432. 37535463

[ref37] Kadivar M.; Aliakbar A. A molecularly imprinted poly 2-aminophenol-gold nanoparticle-reduced graphene oxide composite for electrochemical determination of flutamide in environmental and biological samples. Anal. Methods 2021, 13, 536–551. 10.1039/D0AY01812K. 33449062

[ref38] Atchudan R.; Edison T. N. J. I.; Perumal S.; Vinodh R.; Sundramoorthy A. K.; Babu R. S.; Lee Y. R. Leftover kiwi fruit peel-derived carbon dots as a highly selective fluorescent sensor for detection of ferric ion^†^. Chemosensors 2021, 9, 166 10.3390/chemosensors9070166.

[ref39] Murugan N.; Prakash M.; Jayakumar M.; Sundaramurthy A.; Sundramoorthy A. K. Green synthesis of fluorescent carbon quantum dots from Eleusine coracana and their application as a fluorescence ‘turn-off’ sensor probe for selective detection of Cu^2+^. Appl. Surf. Sci. 2019, 476, 468–480. 10.1016/j.apsusc.2019.01.090.

[ref40] Ham H.; Park N.-H.; Kim S. S.; Kim H. W. Evidence of ostwald ripening during evolution of micro-scale solid carbon spheres. Sci. Rep. 2014, 4, 3579 10.1038/srep03579. 24389995 PMC3880962

[ref41] Verma A.; Singh A.; Chaudhary P.; Tripathi R. K.; Yadav B. C.; Chauhan P.; Kumar D. Photocurrent conversion capability of a 2D WS_2_-polyvinyl alcohol matrix and its DFT-based charge carrier dynamics analysis. Mater. Adv. 2023, 4, 1062–1074. 10.1039/D2MA00962E.

[ref42] Hughes J. P.; Blanco F. D.; Banks C. E.; Rowley-Neale S. J. Mass-producible 2D-WS_2_ bulk modified screen printed electrodes towards the hydrogen evolution reaction. RSC Adv. 2019, 9, 25003–25011. 10.1039/C9RA05342E. 35528637 PMC9069938

[ref43] Bhuvaneswari C.; Palpandi K.; Amritha B.; Paunkumar P.; Lakshmi Priya R.; Raman N.; Ganesh Babu S. Conniving for the first time of BiVO_4_–rGO/CE-BN and its potential as enhanced electrochemical sensing of non-steroidal anti-androgen drug. Microchem. J. 2023, 184, 108174 10.1016/j.microc.2022.108174.

[ref44] Santhosh A. S.; Sahana K. M.; Sandeep S.; Prashanth Kumar P. N.; Alsaiari N. S.; Katubi K. M.; Abualnaja K. M.; Rajabathar J. R. Synthesis and application of a 0D/2D nanocomposite for the nanomolar level detection of an antiandrogen drug. New J. Chem. 2022, 46, 16068–16077. 10.1039/D2NJ01967A.

[ref45] Musuvadhi Babulal S.; Anupriya J.; Chen S. M. Self assembled three dimensional β-Cu_2_V_2_O_7_ hierarchical flower decorated porous carbon: An efficient electrocatalyst for flutamide detection in biological and environmental samples. Chemosphere 2022, 303, 135203 10.1016/j.chemosphere.2022.135203. 35667499

[ref46] Selvi S. V.; Nataraj N.; Chen T.-W.; Chen S. M.; Nagarajan S.; Ko C. S.; Tseng T.-W.; Huang C.-C. In-situ formation of 2H phase MoS_2_/cerium-zirconium oxide nanohybrid for potential electrochemical detection of an anticancer drug flutamide. Mater. Today Chem. 2022, 23, 100749 10.1016/j.mtchem.2021.100749.

