# Supplementary material for: Amplifying Flutamide Sensing through the Synergetic Combination of Actinidia-Derived Carbon Particles and WS2 Platelets
Source: ACS Omega. 2024 Jun 26;9(27):29598–608. doi: 10.1021/acsomega.4c02795 (PMC11238225; doi:10.1021/acsomega.4c02795)
Supplement: Supplementary file 1 — ao4c02795_si_001.pdf [file ao4c02795_si_001.pdf]

SUPPLEMENTARY DATA

Amplifying flutamide sensing through the synergetic combination of *Actinidia*-derived carbon particles and WS_2 platelets

Yiran Luo[1], P. Rupa Kasturi[1], Tara N. Barwa[1], Eithne Dempsey[1,2], Carmel B. Breslin[1,2*]

1 Department of Chemistry, Maynooth University, Maynooth, Co. Kildare, Ireland
2 Kathleen Lonsdale Institute, Maynooth University, Maynooth, Co, Kildare, Ireland

*Corresponding Author: Carmel.Breslin@mu.ie

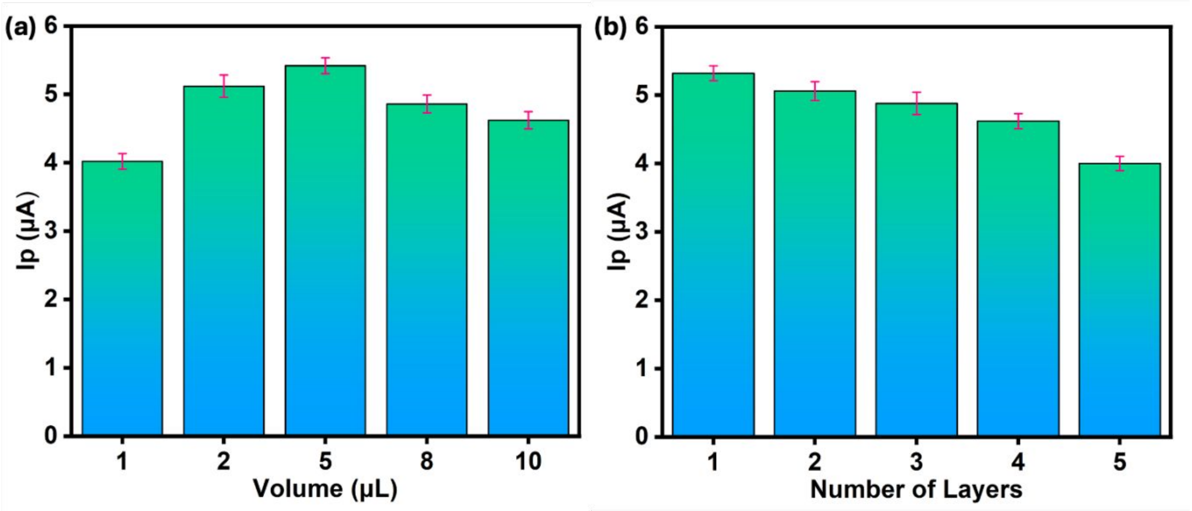

Figure S1: (a) Influence of the drop casting volume of the CPs-WS_2 dispersion and **(b)** the number of 5 μL layers applied on the peak current recorded during the reduction of 100 μM FLD.

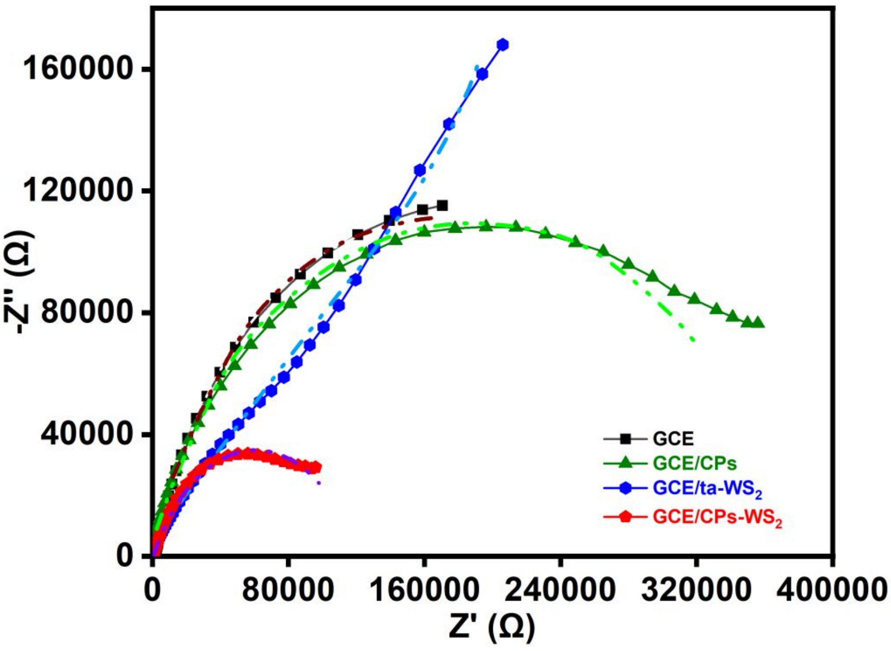

Figure S2. EIS spectra recorded for GCE, GCE/CPs, GCE/WS_2 and GCE/CPs-WS_2 at −0.68 V in 100 μM FLD in phosphate buffer. A Warburg diffusional element was added to the Randles Cell circuit for GCE/ta-WS_2. The GCE, GCE/CPs, GCE/CPs-WS_2 were all modelled with the Randles Cell.

Table S1. Peak currents recorded for different concentrations of FLD in 0.1 M phosphate buffer over a 15-day period. Average value obtained from three experiments (n = 3).

FLD (μM)	First Day Average Current (μA)	After 10 days Average Current (μA)	After 15 days Average Current (μA)	%RSD
10	2.86	2.83	2.47	5.64
30	4.07	4.03	3.87	1.87
50	5.24	5.12	5.04	1.38
100	8.24	8.12	8.02	0.95

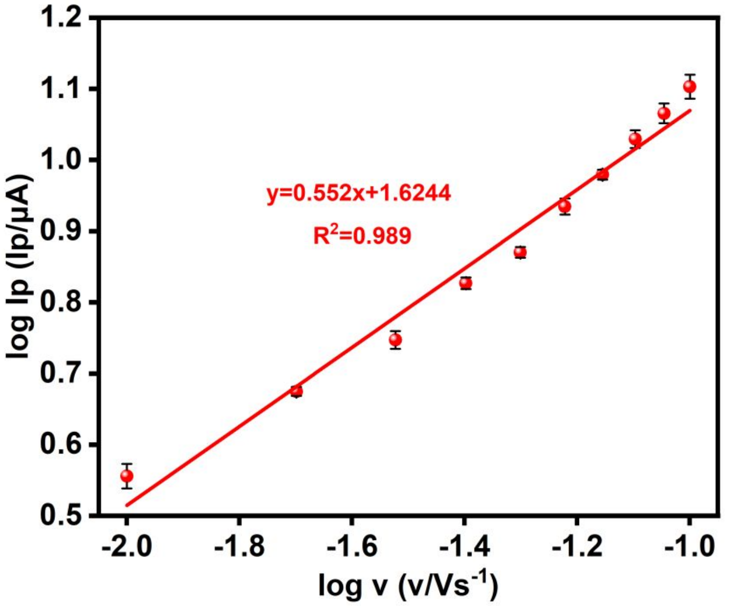

Figure S3. Logarithm of the peak current plotted as a function of the logarithm of the scan rate for 100 μM FLD in phosphate buffer.

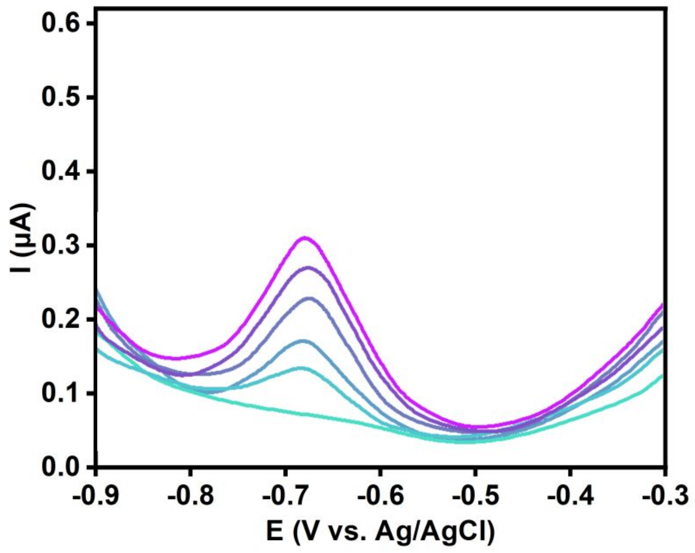

Figure S4. DPVs recorded for FLD concentrations ranging from 1 nM, 5 nM, 10 nM, 20 nM and 30 nM (blue trace without a peak is the background electrolyte).

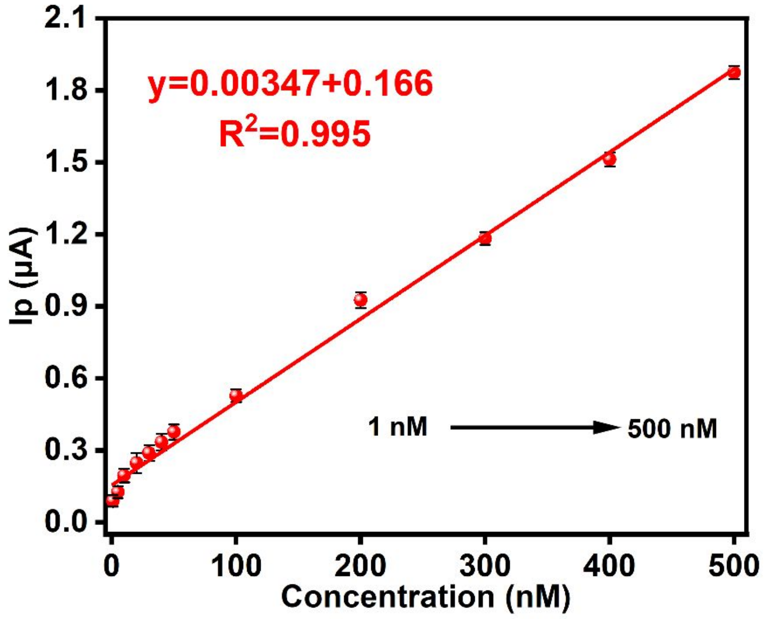

Figure S5. Calibration curve plotted with the DPV peak currents against the FLD concentration from 1 nM to 500 nM.

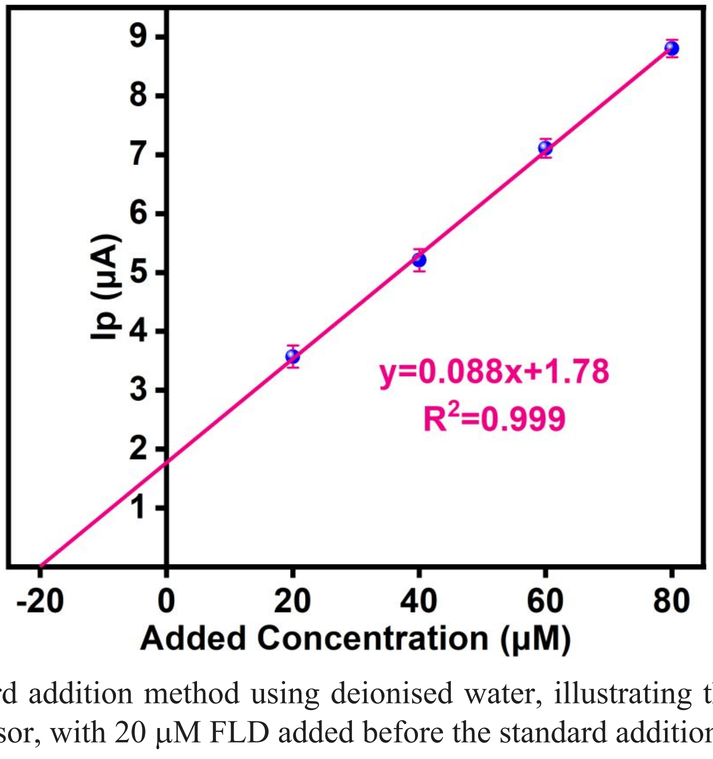

Figure S6. Standard addition method using deionised water, illustrating the accuracy of the GCE/CPs-WS_2 sensor, with 20 μM FLD added before the standard additions were made.
